# Phagocytosis in Dental Lesions

**Published:** 1891-05

**Authors:** F. W. Sage

**Affiliations:** Cincinnati, O.


### Phagocytosis in Dental Lesions.

BY F. W. SAGE, D.D.S., CINCINNATI, O.

Read before the Mississippi Valley Dental Society, held at Cincinnati, March, 1891.

It is the object of this paper to review somewhat in detail two papers published in the January number of the *Dental Advertiser*, the titles of which are "Phagocytosis," and "The Mechanism of Infection," the authors respectively being Dr. Frank W. Low, of Buffalo, N. Y., and Dr. Bouchard, of Paris.

Phagocytosis is fully defined by Dr. Low; without presuming too much on our hearers not having read the article referred to, we venture to present in the briefest terms his definition. A "phagocyte," then, is a cell, which possesses the power in a greater or less degree of appropriating and devouring solid particles. He names five tissues in which these phagocytes or amœboid cells abound; as we shall have in this paper to consider more particularly the invasions of the pyogenic microbe, we shall here name one only—the white corpuscles of blood and mucus.

Phagocytosis is, then, according to the definition, and for our present purpose, an exhibition (in that interesting phenomenon commonly known as suppuration) of the battle going on between the powers that waste and the forces that tend to prevent waste of tissue. The microbe is the invader, seeking to break down and lay waste; the phagocyte is the watch dog seeking to destroy the invader.

Notwithstanding Dr. Low's admirable presentation of the details of various experiments made by way of attesting the truth of a theory he advances, he seems to the writer to have fallen short of what he has attempted. He says:

"Where teeth on the one hand remain in the jaw after treatment, they shortly become, apparently, perfectly healthy, (a condition that could not obtain so long as pyogenic micro-organisms were present in the tissue); on the other hand, those having been first extracted and then experimentally treated, have almost, if not quite, invariably developed bacteria cultures. Having in mind these facts, but one inference is rationally to be drawn, namely, that the phagocyte cell acts as the factor of elimination, devouring all bacteria, both in the pulp canal and in the tissue about the apex of the root." And then after calling attention to Dr. Miller's assurance of the inadequacy of any and all agents at present employed, to effect a perfectly aseptic condition of tooth-roots, and having also directed attention to the success attending the sole use of dry hot air and simple mechanical cleansing of the roots before filling, he adduces the following experiment: an upper molar, having putrescent pulp canals, was extracted, suitable precaution being at once taken against the possible ingress of pathogenic microbes, after which the pulp chamber and canals were carefully treated by the "immediate root process" (I quote his words). The roots were then split open and immersed in agar-agar. Almost immediately, the anterior buccal root, which he states *had not been filled*, yielded a colony of cocci. The filled roots however gave no such result.

The writer admits his inability to see that this experiment illustrates the truth of Dr. Low's statement already quoted, that teeth having been *first* extracted and *then* experimentally treated, have almost if not quite, invariably, developed bacteria cultures. The result of the experiment seems to establish the exact opposite fact. It is, however, a matter of no little moment that if the "immediate treatment process" employed (again I quote his words) was according to the method of using simply dry hot air and mechanical cleansing, to which he referred, the success of the method of procedure seems well enough established.

It is unfortunate that Dr. Low does not explicitly state what he meant by the "immediate treatment process"; still, in view of what he has said of the success of the "dry air and mechanical cleansing process," it seems reasonably safe to assume that he used no medicaments. And yet in view of what we shall presently attempt to consider, it is not essential to our purpose that we shall insist on the assumption that he used no medicaments. The result is in any event interesting as indicating the fact that whatever disturbances may follow the "immediate root" process we should direct attention to the apical space.

Dr. Low has distinctly declared that no cultures developed in the two filled roots. Again referring back to his statement that "the phagocyte cell acts as the factor of elimination, devouring all bacteria both in the pulp canal and in the tissues about the apex of the root," we say that the result of his experiment seems to plainly indicate that in the case of the unfilled root this inhibitory action had not taken place. It is not then unreasonable to assume that neither had it taken place in the other two roots so successfully filled.

We point out the significant results of his experiment, not in any spirit of cavil: the valuable results incidentally shown—of the "immediate root process"—are of sufficient interest to us, for present consideration, and call for an expression of gratitude to be tendered Dr. Low. It is worthy of passing comment, at least, that this single experiment of Dr. Low's seems to refute Dr. Miller's statement that "beyond all doubt none of the agents now employed to bring about a perfect asceptic condition of tooth roots, are equal to the emergency." The context shows that Dr. Low recognized the full significance of the experiment in this particular respect. It would seem that Dr. Miller did not have in mind dry hot air and mechanical cleansing, if we are right in assuming that upon them alone Dr. Low relied in his experiment.

We find on following Dr. Low, further on, that he assumes that his readers must regard with increduility the idea of the phagocyte's penetrating the dentinal tubules, or even the foramen of the pulp canal. Why he should assume such incredulity we are at a loss to understand, unless it be as regards the dentinal tubules only, for it seems to be implied in the quotation which we are about to give, that the phagocyte is capable of reaching any point to which the blood has access. We again quote: "The writer would venture the opinion that when stasis is complete, the battle goes against the phagocyte, because reinforcements can no longer come to the rescue, while the bacteria proliferate without further hindrance, receiving new colonies with the impactment of each morsel of decomposing vegetable or animal matter that lodges in the cavity of decay."

Leaving at this point the special consideration of Dr. Low's paper, and preserving in mind only the fact which he seems to have demonstrated that roots may be at once rendered asceptic by simple means, we find before us a field for interesting speculation as to what takes place in the "always present abscess"* at the apex of the tooth or beyond that point, after the filling has been inserted. At the risk of rehearsing what is already trite, the writer refers to a time when the most heroic treatment for alveolar abscess was the only kind of treatment to which any reference whatever was made, by at least one author whose work is to-day in the hands of every dental student. That was the total eradication of the abscess by an operation directed through the alveolar wall. About seven years ago the writer of this had suggested to him—through his observation of the result of empirical practice on his part and on the part of other practitioners—the feasibility and the expediency of filling diseased roots after cleansing and disinfecting merely, and oftentimes without attempting to reach the abscess. That is to say, he discovered that in the cases of individuals of certain temperaments, no unpleasant reaction followed this modified course of treatment. In view of the latest discoveries in bacteriology, it will be seen that this was a leap in the dark. At this day, in the light of recent revelations whereby we are enabled to identify and classify not only the various pathogenic microbes, but also their several foes lying in wait to guard the tissues against their inroads, we seem almost to have reached a stage when it shall be possible to define such hitherto vague terms, as diathesis, vital resistive force, and *vis medicatrix naturæ.* The florid complexion, red hair, bulbulous nose, of the individual of an inflammatory diathesis, seems already to suggest something better than the vague term "predisposition;" it calls for the identification of the special microbe producing the specific disease, and at the same time leads us to the naming of the particular phagocyte, the absence of which allows of the proliferation of the mischief-breeding microbe.

It is interesting to recall various small items which a few years ago were significant as *causes* of inflammation, although they fell short of supplying in explicit terms the why and the wherefore of their being causes. There was the "infected broach," which being inserted into the root canal, provoked the quiescent abscess to renewed activity, or, on the assurance that the broach used had been carefully sterilized, irritation was attributed to the forcing through the foramen of effete matter already contained in the root. No one seems to have discovered a solution of what was, after all, a mystery attending this matter, notwithstanding Lister long ago dropped a hint which might have been appropriated by the dental profession, in a suggestion made that the lance should be applied to abscesses in the soft tissues, only after having drawn the integuement obliquely aside, so that on its return after being released, the air might be excluded. In view of what Dr. Bouchard has to say regarding the phagocyte and the bactericidal condition, it will not be difficult to account for the fact that the infected broach, in the hands of the most reckless operator, does not invariably excite inflammation. For an explanation of this we refer the author to his admirable paper.

But to return to the inquiry, "What takes place beyond the apex after the root has been filled?" To discuss this question exhaustively would take more time than the writer feels is properly alloted to him; he will, therefore, call attention to only a few features of what seems reasonably well established. We are continually tempted to turn aside, lured by the curious use of terms in vogue only a decade ago—"laudable pus," "decomposition of pus recurring in secondary inflammation," and so on.

But to resume: the treatment of alveolar abscess in years past has been founded on a principle of reasoning from effect to cause, without an intelligent apprehension of the nature of either the effect or the cause. We argued the necessity of stopping the root canal after a course of treatment in order to obliterate, or do away, with a reservoir into which fluids might seep, there to decompose and "react" upon the parts beyond. The condition thus brought about was one in which, in popular parlance, the lymphatics could have an opportunity to control, absorb, or what not, the seeping fluids. In all this there was no recognition of what Dr. Bouchard terms the bactericidal condition, accessory to the aggressive function of that microbe destroyer, the phagocyte. We knew that through the use of medicaments we could indure, sooner or later, a condition in the abscess, of tolerance, which we recognize as a restoration of the balance between the so-called vital resistive force and the disease-breeding agents. But that this was a chemical condition slowly brought about, through which microbes of infection were destroyed, we had no suspicion. We viewed the recurrence of swelling in the gum months and even years after our treatment of roots, noting the absence of pain, but being unable to account for that fact. There the microbes, according to Dr. Bouchard's theory, were again at work, but with weakened functions, owing to the fact of their having to contend with the vaccine elements of other colonies of microbes once occupants of the field. Here again we recall a principle laid down in works on surgery: "That a part once inflamed becomes weakened, inviting a recurrence of inflammation on the slightest exposure to the causes of inflammation." The recurring inflammation may be persistent, but it is of the low grade indicated by the word "chronic." The microbe is persistent because through the prevention of vascular dilatation, exudation and diapedesis the phagocyte is afforded no opportunity to combat him. At the same time, as Dr. Bouchard affirms, the microbe's functions are embarrassed from the fact that while on the one hand it secretes noxious elements favorable to its own development, it also secretes these vaccine elements before referred to, the protective influence of which presently begins to be manifested.

In this desultory way we believe we have gone over the principal ground of research in what most concerns us as dentists to know, as regards the action of microbes in alveolar abscess at least.

The abscess then, unless extirpated by a heroic operation, usually remains in a quiescent state, subject to the influence of after-inflammation.

